# 

**Published:** 1900-02

**Authors:** 


### Society Notes.

### The Central Texas Medical Association.

The Central Texas Medical Association will meet the second Tuesday and Wednesday in July. The meeting will be held in Waco. The following officers were elected for the ensuing year:

President, Dr. A. C. Scott, Temple.

First Vice-President, Dr. A. M. Curtis, Waco.

Second Vice-President, Dr. J. W. Hunter, Waco.

Secretary and Treasurer, Dr. N. A. Olive, Waco.

Chairman of Section of Surgery, Dr. W. C. Blalock, Kosse.

Chairman of Obstetrics and Gynecology, Dr. A. R. Kuykendall, Morgan.

Chairman of Ophthalmology and Otology, Dr. J. W. Woodson, Temple.

Chairman of Section of Medicine, Dr. Embree, of Temple.

Section of Diseases of Nervous System and Medical Jurisprudence, Dr. John S. Turner, San Antonio.

### Texas State Medical Association.

SAN ANTONIO, TEXAS, Feb. 2, 1900.

*To the Members of the Texas State Medical Association:*

GENTLEMEN: The State Medical Association will meet in Waco April 24. It is the intention of the Secretary to issue the program not later than March 15. Inasmuch as it will be impossible to reach all by letter who may wish to contribute papers to the Section on Surgery, I take this means to invite those who may wish to present papers to this section to send titles thereof to the Secretary, Dr. J. B. Stuart, Houston, Texas, or to myself, before that date.

Fraternally yours,

B. F. KINGSLEY,

Chairman Section on Surgery.

**Dyspnoea of Fibroid Phthisis.**—

| | | | |
|---|---|---|---|
| ℞ | Sodii chloridi | 10 | gm. |
| | Sodii bromidi | 5 | " |
| | Sodii iodidi | 1.5 | " |
| | Aquæ destil | 100 | " |

M. S. One to three teaspoonfuls daily in milk.—*Potain.*

